# The Application of Awake-Prone Positioning Among Non-intubated Patients With COVID-19-Related ARDS: A Narrative Review

**DOI:** 10.3389/fmed.2022.817689

**Published:** 2022-02-07

**Authors:** Lingli Chen, Yan Zhang, Yi Li, Chao Song, Fengyu Lin, Pinhua Pan

**Affiliations:** ^1^Center of Respiratory Medicine, Xiangya Hospital, Central South University, Changsha, China; ^2^Hunan Engineering Research Center for Intelligent Diagnosis and Treatment of Respiratory Disease, Changsha, China; ^3^National Key Clinical Specialty, Branch of National Clinical Research Center for Respiratory Disease, Xiangya Hospital, Central South University, Changsha, China; ^4^Department of Infection Control Center, Xiangya Hospital, Central South University, Changsha, China; ^5^National Clinical Research Center for Geriatric Disorders, Xiangya Hospital, Changsha, China; ^6^Center of Respiratory Medicine, Xiangya Hospital, Central South University, Changsha, China

**Keywords:** COVID-19, SARS-CoV-2, ARDS, awake prone position, non-invasive respiratory support

## Abstract

The coronavirus disease (COVID-19) pandemic has significantly increased the number of patients with acute respiratory distress syndrome (ARDS), necessitating respiratory support. This strain on intensive care unit (ICU) resources forces clinicians to limit the use of mechanical ventilation by seeking novel therapeutic strategies. Awake-prone positioning appears to be a safe and tolerable intervention for non-intubated patients with hypoxemic respiratory failure. Meanwhile, several observational studies and meta-analyses have reported the early use of prone positioning in awake patients with COVID-19-related ARDS (C-ARDS) for improving oxygenation levels and preventing ICU transfers. Indeed, some international guidelines have recommended the early application of awake-prone positioning in patients with hypoxemic respiratory failure attributable to C-ARDS. However, its effectiveness in reducing intubation rate, mortality, applied timing, and optimal duration is unclear. High-quality evidence of awake-prone positioning for hypoxemic patients with COVID-19 is still lacking. Therefore, this article provides an update on the current state of published literature about the physiological rationale, effect, timing, duration, and populations that might benefit from awake proning. Moreover, the risks and adverse effects of awake-prone positioning were also investigated. This work will guide future studies and aid clinicians in deciding on better treatment plans.

## Background

The coronavirus disease (COVID-19) pandemic has increased the number of patients with hypoxemic respiratory failure due to severe acute respiratory syndrome coronavirus 2 infection. Acute respiratory distress syndrome (ARDS) is responsible for the lung injury characterized in COVID-19 patients (1). The incidence of COVID-19-related ARDS (C-ARDS) in hospitalized patients is around 14–30% (2). Approximately 5% of these patients require mechanical ventilation support and further intensive care unit admission (3). The shortage of mechanical ventilators and the heavy burden on the intensive care unit workload may contribute to increasing mortality, therefore prompting clinicians to explore an alternative, simple, and effective strategy during the COVID-19 pandemic.

Previous clinical evidence has shown that invasive or non-invasive mechanical ventilation (e.g., BIBAP and CPAP) with adjunct prone positioning could improve clinical outcomes for hypoxemic patients. For instance, a prospective randomized controlled trial determined that prone positioning almost halved 28-day and 90-day mortality in patients with severe ARDS caused by a variety of etiologies receiving invasive mechanical ventilation (4). This corroborates well with the findings of a recent metaanalysis (5) and a Cochrane systematic review (6) which revealed that patients in prone positioning respond well to invasive mechanical ventilation, improving oxygenation levels, and reducing mortality. This finding led to prone positioning being recommended in international guidelines for the management of COVID-19-related ARDS. Meanwhile, the WHO advocates 12–16 h/day of prone positioning for moderate-to-severe C-ARDS patients (7). Besides, the Surviving Sepsis Campaign panel recommends that C-ARDS patients with mechanical ventilation should be managed similarly to patients with typical ARDS (8). Furthermore, recent guidance by the Intensive Care Society (ICS) also recommends awake-prone positioning for all suitable COVID-19 patients (9). These recommendations were extrapolated from physiological principles and clinical evidence obtained in patients with typical ARDS undergoing invasive mechanical ventilation, distinct from the C-ARDS population (10).

### Search Strategy

To include maximum published studies regarding awake-prone positioning, electronic databases such as PubMed, Embase, Medline, Cochrane Central Register of Controlled Trials, and WHO SARS-CoV-2 Research Database were explored from inception to December 17, 2021. The key search terms were defined as follows: (i) awake-prone position or awake self-prone or awake pronation or awake-prone positioning; (ii) acute respiratory distress or ARDS or hypoxemic respiratory failure; (iii) coronavirus 2019 or COVID-19 or SARS-CoV-2. This study was only evaluated literature in English.

## The Clinical Features Of Typical And Covid-19-Associated ARDS

Typical ARDS is characterized by non-cardiogenic pulmonary edema, refractory hypoxemia, and reduced aerated lung size, which indicates low respiratory compliance (11). C-ARDS is a specific disease somewhat consistent with typical ARDS, including inflammatory edema, resulting in different levels of lung collapse and eventually leading to severe ventilation–perfusion mismatch and shunts. Massive lung microthrombosis develop, resulting in various degrees of dead space and insufficient hypoxemia (12). Unlike typical ARDS, C-ARDS typically appears as ground-glass opacities in the bilateral lungs on CT and X-ray imaging (13). Interestingly, lung compliance may be normal or reduced in C-ARDS, with a mismatch between severe hypoxemia and compliance possibly causing ventilator-associated or patient self-inflicted lung injury (P-SILI). In addition, the typical “cytokine storm” and higher cytokine levels seen in classical ARDS are seldom observed in C-ARDS (14). Gattinoni et al. described two types of C-ARDS, the L and the H phenotypes (15). However, it is widely accepted that COVID-19-induced pneumonia cannot be strictly divided into two phenotypes exhibiting marked phenotypic diversity (16).

### The Physiological Rationale of Prone Positioning

**Figure 1:**
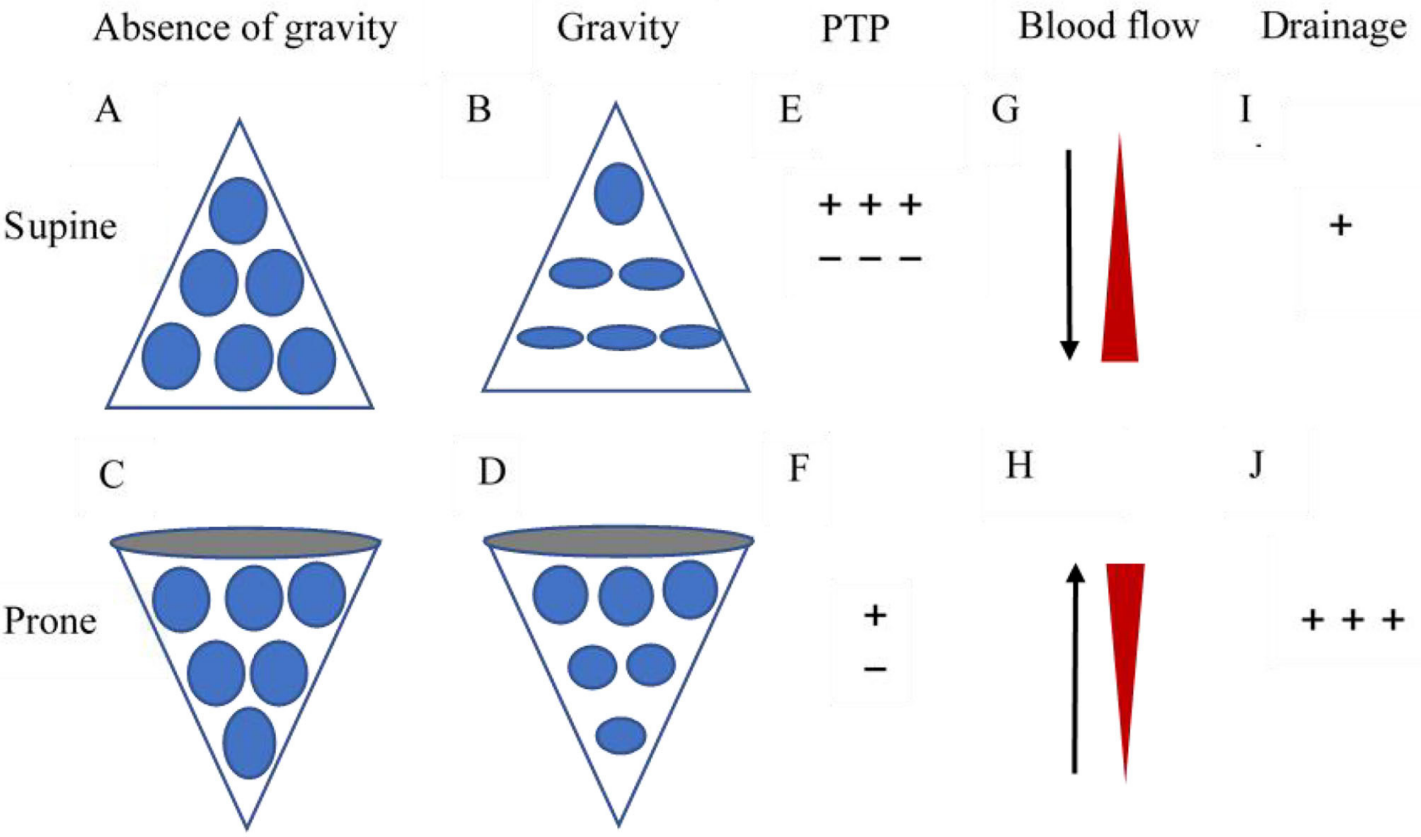
Schematic diagram of the pathophysiological mechanism of prone position treatment in ARDS. **(A)** and **(C)** represent the natural form of alveoli without the effect of gravity. **(B)** Under the gravitational gradient and pleural pressure, the alveolar volume in the dorsal area is significantly smaller than that in the ventral area, thus the changes caused by ARDS will be more significant. **(D)** In the prone position, the effect of the gravitational gradient or pleural pressure is reversed, i.e., the volume of the ventral alveoli decreases while that of the dorsal alveoli increases. **(E)** In the supine position, the ventral transpulmonary pressure (PTP) significantly exceeds the dorsal PTP. **(F)** Prone positioning reduces the difference between the ventral and dorsal PTP, making ventilation more homogeneous. **(G)** The previously dependent lung continues to receive the majority of the blood flow as alveoli reopen. **(H)** The newly dependent lung continues to receive the minority of the blood flow as alveoli begin to collapse. **(I)** In the supine position, the bronchial drainage is limited. **(J)** Prone positioning improves bronchial drainage.

Prone positioning may have numerous beneficial effects on the lung physiology of ARDS. From the physiological rationale point of view, prone positioning plays a pivotal role in reducing ventilation/perfusion mismatch, intrapulmonary shunt, and recruiting aerated lung regions (17). The schematic diagram of the pathophysiological mechanism of prone position treatment in ARDS was described by Gattinoni et al. (18) (Figure 1). It was established that the supine position might be detrimental to lung function, particularly in overinflation of the ventral alveoli and atelectasis of the dorsal alveoli. This is due to increased transpulmonary pressure gradient and compression of alveoli secondary to direct pressure from the mediastinum. Moreover, vessels with poor ventilation but high perfusion affected by the gravitational gradient in the supine position results in ventilation/perfusion mismatch (19). In contrast to the supine position, prone positioning could improve ventilation–perfusion mismatch by decreasing ventral alveolar overdistention and dorsal alveolar collapse, further ameliorating the ventral–dorsal transpulmonary pressure gradient. Besides, it generates a more homogeneous distribution of gas, thereby enhancing the recruitment of dorsal lung segments. Considering the higher density of pulmonary vessels in the dorsal lung region, which is independent of gravitational factors, there is limited compression of the dorsal regional lung with the maintenance of dorsal perfusion while the prone position maintains the perfusion pattern relatively constant. Subsequently, a reduction in intrapulmonary shunting is observed (20). Additionally, the recruitment of collapsed alveoli in the posterior lung regions remains functional, alleviating hypoxemia. Prone positioning may also facilitate the drainage of respiratory secretions from the dorsal lung regions (17). The contraction of the diaphragmatic muscle may exert a more uniform distribution when facing the open dorsal lung during the prone position, whereas it exerts additional localized stress when facing the collapsed lung during the supine position (21). Therefore, prone positioning can evenly distribute the mechanical force from the ventilator to the entire lung during inhalation and reduce lung damage. It may have synergistic lung-protective effects with low tidal volume ventilation.

Due to its positive physiological effects, prone positioning was investigated in spontaneously breathing patients. Similar to intubated ARDS patients, spontaneous breathing can produce high respiratory drive and forceful inhalation efforts, inducing lung injury akin to ventilator-induced lung injury (VILI) or P-SILI. Esnault et al. (22) enrolled patients with mild to severe ARDS caused by COVID-19, including intubated and mechanically ventilated patients with spontaneous breathing in the supine position, and identified that P0.1, defined as the negative pressure measured at the airway opening, was frequently above 3.5 cm H2O, suggesting high neural respiratory drive. A prospective study revealed that the respiratory rate decreased in awake patients during prone positioning, thereby indicating that pronation may decrease the inspiratory effort (23). Another benefit of the prone position in infants with severe bronchiolitis requiring non-invasive ventilation is that the position considerably decreases inspiratory efforts and neural drive to the diaphragm (24). Thus, prone positioning may prevent or reduce secondary lung injury caused by an increased respiratory drive due to compensatory hypoxia. The assessment of respiratory drive and inspiratory efforts are essential in understanding the impact of prone positioning in patients with spontaneous breathing.

### Evidence of Awake-Prone Positioning in Typical ARDS

While prone positioning is an evidence-established practice in patients with typical ARDS receiving invasive mechanical ventilation (IMV), evidence of its role in spontaneously breathing patients is scarce and mostly collected from small observational or retrospective studies. In 2015, Scaravilli et al. (25) performed a retrospective study in 15 non-intubated patients receiving oxygen or non-invasive ventilation modalities for hypoxemic respiratory failure and theorized that the repeated prone positioning procedure is feasible and proved that prone positioning improves oxygenation levels in awake patients, although this effect is transient. Valter et al. (26) reported that four patients who underwent awake-prone positioning rapidly improved their oxygenation levels and avoided intubation. Feltracco et al. (27) showed that five lung transplant patients recovering from hypoxemia receiving non-invasive ventilation in the awake prone positioning. Recently, Ding and colleagues (28) reported on a multicenter prospective cohort study of 20 non-intubated patients with moderate to severe ARDS, needing High Flow Nasal Cannula (HFNC) or non-invasive ventilation (NIV) support, who underwent an average of 2-h of prone positioning twice daily. The study found that using early prone positioning combined with HFNC/NIV increased PaO_2_/FiO_2_ by 25 to 35 mmHg compared with conventional oxygen therapy and that the application of prone positioning may cut the need for intubation by up to half of the patients. However, 9 patients (45%) with ARDS were eventually intubated, signaling that awake-prone positioning may result in a higher risk of delayed intubation.

### Evidence of Awake-Prone Positioning During the COVID-19 Pandemic

The impact of awake-prone positioning on clinical outcomes, intubation rate, and survival rate in patients with COVID-19 remains undetermined. Although some studies have shown negative results (29–32), most of the early research denoted that prone positioning may improve short-term oxygenation levels with no severe complications (Supplementary Table 1). Specifically, in a prospective cohort study with 56 patients, Coppo et al. (33) theorized that prone positioning was feasible and effective in substantially improving oxygenation levels in awake patients with C-ARDS, and the effects were maintained after resupination in half of the patients. Similarly, Thompson and colleagues (34) carried a single-center cohort study and reported that awake-prone positioning improved peripheral oxygen saturation (SpO2) compared with baseline, and only 7 (37%) patients required intubation. Sartini and colleagues (35) performed a cross-sectional before-after study of 15 awake patients with mild to moderate ARDS undergoing NIV in the prone position for 3 h. On the first day, SpO2 and PaO2:FIO2 improvements were sustained 60 min after pronation in 80% of the patients. At the 14-day follow-up, 9 patients were discharged. Caputo and colleagues (36) described their experience in an observational cohort study of 50 patients in the emergency department. After 5 min of awake-prone positioning, SpO2 improved from 80 to 94%. However, 13 patients (24%) failed to improve or maintain oxygen saturations and required endotracheal intubation within 24 h of arrival to the emergency department (ED). In a retrospective study by Xu and colleagues (37), 10 patients with COVID-19 and PaO2/FiO2 <300 mmHg were placed early in the awake prone position and combined with HFNC for more than 16 h per day. They reported that PaO2/FiO2 was significantly elevated, and none of the patients progressed to critical condition or needed endotracheal intubation. In another retrospective study of 10 patients by Ng and colleagues (38), 9 patients were successfully weaned off of oxygen. As expected, these small-scale observational studies provide clues as to how awake-prone position may be a therapeutic strategy for patients with COVID-19.

Presently, there is no strong evidence to support the use of awake-prone positioning in patients with COVID-19. Randomized clinical trials investigating the clinical efficacy of awake-prone positioning with various non-invasive approaches supporting patients with acute hypoxemic respiratory failure are lacking, but inspired by a randomized, controlled, multinational, open-label metatrial (39). Herein, 1,121 patients were enrolled and randomly divided into an awake-prone positioning (*n* = 564) group and a standard care (*n* = 557) group. The results revealed that the treatment failed in 223 (40%) out of 564 patients assigned to the awake-prone positioning group and in 257 (46%) of the 557 patients in the standard care group (relative risk 0.86 [95% CI 0.75–0.98]). The hazard ratio (HR) for intubation was 0.75 (0.62–0.91) and the HR for mortality was 0.87 (0.68–1.11) with awake-prone positioning compared with standard care within 28 days of enrolment. Besides, the incidence of prespecified adverse events was minimal and similar in both groups. These results indicated that awake prone positioning in patients with COVID-19-induced hypoxic respiratory failure reduces the incidence of treatment failure and intubation without adverse effects, and also supported its routine use in patients who receiving HFNC treatment.

Prone positioning is believed to be compatible with both NIV and HFNC. Although both modalities are beneficial to patients with ARDS (40, 41), they operate *via* different mechanisms. On one hand, NIV applies positive end-expiratory pressure (PEEP), which can reduce atelectasis and promote alveolar recruitment by increasing functional residual capacity and reopening the collapsed alveoli, thereby improving the ventilation–perfusion matching and shunt. On the other hand, HFNC reduces breathing effort and assists respiratory muscles during inspiration. The resulting reduction in the anatomical dead space improves oxygenation levels (28). However, NIV and HFNC may simultaneously result in overdistension in the previously well-ventilated alveoli and insufficiently address the hypoxemia secondary to ARDS (42). Similarly, patients with ARDS can generate high respiratory drives during spontaneous breathing, and the ensuing powerful inspiratory efforts can increase the risk of P-SILI. In this context, prone positioning appears to prevent P-SILI by decreasing respiratory efforts and regional hyperinflation (43, 44).

### Potential Adverse Events and Drawbacks of Awake-Prone Positioning

Not all patients can be placed in the prone position since the latter is affected by various factors, including age, cognitive impairment, fatigue, complications, comfort, and disease status, among others. The majority of patients with hypoxemic respiratory failure with spontaneous breathing or undergoing NIV/HFNC can tolerate the awake-prone positioning. Nevertheless, a minority will develop mild complications such as back pain, vomiting, etc. (Table 1). Overall, awake-prone sessions are relatively shorter than the 12–16 h recommended with IMV, partly due to limited patient tolerance. Despite not substantially improving long-term clinical efficacy in patients with C-ARDS, awake-prone positioning improves short-term oxygenation levels and decreases respiratory rate among patients tolerating the sessions. Several early randomized clinical trials of spontaneously breathing ARDS patients placed in prone positions did not show any clinical benefit compared with standardized care. Ferrando et al. (31) reported that the use of awake-PP as adjunctive therapy to HFNO did not prevent intubation and the 28-day mortality risk was not affected compared to the use of HFNO alone. Likewise, Elharrar et al. (32) postulated that prone positioning improved oxygenation levels in only 6 (25%) out of the 25 participants and was not sustained in half of those after resupination. Furthermore, Coppo et al. (33) observed no difference in the incidence of tracheal intubation between responders and non-responders in their cohorts. Although these studies may be limited by the late use of the prone position and shorter duration, it should be noted that the effects of the prone position were transient and often reverted to baseline after resupination.

**Table 1:** Potential adverse events in the awake prone position.

**Pressure sores (e.g., facial)**
**Nerve compression (e.g., brachial plexus injury)**
**Crush injury**
**Venous stasis (e.g., facial edema)**
**Diaphragm limitation**
**Dislodging vascular catheters or drainage tubes**
**Retinal damage**
**Transient reduction in arterial oxygen saturation**
**Vomiting**
**Transient arrhythmias**

Besides some adverse events and no long-term clinical benefits, the main risk of awake-prone positioning remains an undue delay in intubation. This could potentially exacerbate lung injury and worsen prognosis as delayed intubation correlates with increased mortality in ARDS patients (31, 33, 45). However, the optimal timing of intubation and mechanical ventilation for ARDS patients is unclear. Early initiation (<24 h of HFNC use) of APP in acute hypoxemic respiratory failure secondary to COVID-19 improves 28-day survival (46). Interestingly, Cammarota et al. (30) employed ultrasound to evaluate the effects of awake-prone positioning on diaphragmatic thickening fraction in patients on non-invasive ventilation. Diaphragmatic thickening fraction reflects the magnitude of diaphragmatic effort and may predict successful weaning of mechanical ventilation (47). The study involved 20 patients and reported that despite improving peripheral blood oxygen saturation, the prone position reduced the comfort score from 7.0 (6.0–8.0) to 6.0 (5.0–7.0) and increased the diaphragmatic thickening fraction from 33.3% (25.7–40.5%) to 41.5% (29.8–50.0%). Therefore, the application of the awake-prone position improved the oxygenation levels at the expense of a greater diaphragmatic thickening fraction. Thus, more research involving close monitoring of diaphragmatic electromyogram or lung ultrasound is warranted.

### Protocols of Awake-Prone Positioning

There are currently no uniform criteria for the application of awake-prone positioning in patients with hypoxemic respiratory failure. Many factors are yet to be assessed, such as determining the appropriate patients, the optimal frequency, duration, and the weaning time. The duration of prone positioning varies in different studies, but the overall aim is to maintain the prone position as long as possible, ideally 16 h or more per day. Ehrmann et al. (39) carried out a randomized trial and noted that 25 (17%) out of 151 patients in the prone position for at least 8 h failed the treatment. Conversely, 198 (48%) out of 413 patients in the prone position for less than 8 h had treatment failures similar to the overall treatment failure rate of the control group (46%). Furthermore, an analysis of three larger studies in Mexico (*n* = 430), France (*n* = 402), and the United States (*n* = 222) demonstrated contrasting effects for different durations in the prone position. Patients from Mexico remained in the prone position (average of 9 h) the longest and experienced the greatest effects. On the other hand, patients from France (average prone of 2.9 h) and the United States (average prone of 4.4 h) experienced lower effects. Sustaining the awake-prone position for an extended period may correlate with improvements in oxygenation levels, whereas the benefits of very short sessions may be questionable. The latest meta-analysis illustrated that prone positioning can improve the oxygenation level amongst non-intubated patients with acute hypoxemic respiratory failure when applied for at least 4 h daily (48). Most studies (Supplementary Table 1) applied the protocol of proning for either ≥3 h twice daily, 10–12 h, or more than 16 h (29, 31–35, 37, 39, 49–52). However, there are no published studies to confirm the optimal frequency and duration for awake-prone positioning. Moreover, tolerance may become a limitation to the treatment duration.

Patient compliance plays a crucial role in tolerating awake-prone positioning, especially for patients with obesity, pregnancy, bloating, etc. To improve patient compliance, additional pillows or rolled sheets and towels may be required to increase comfort and relieve pressure on bony prominences. New support equipment has also been developed to reduce discomfort. Some studies indicate that prior education regarding awake-prone positioning (e.g., handouts) may also help improve compliance (53). Reverse Trendelenburg position (9), Rodin's position (54), the dolphin prone position (55), and alternating prone positioning (56) may assist in comfort and are intended to be tolerated by patients who are unable to undergo prone positioning (Table 2). However, these alternative positions need to be evaluated in randomized controlled trials since the evidence is still scarce.

**Table 2:** The implementation protocol of different alternative positions.

**Rodin's thinker**	**The dolphin position**	**Reverse Trendelenburg position**	**Alternating prone positioning**
Patients sit on a chair and rest their chest on a flat, elevated surface (i.e., their bed or a desk, at an intermammillary line), thus placing the chest in a “semi-prone” position. The head is laid on the arms, elevated and crossed.	Patients reverse their position on the bed, placing their head in the “bed foot area.” In this way, the joint of the bed, normally dedicated to the inclination of the lower limbs, is used to achieve a comfortable chest position.	In a supine position, the patients' hip and knees are not flexed, but the head and chest are elevated at 30° to the abdomen and legs.	1.30 min−2 h: lying on your belly 2. 30 min−2 h: lying on your right side 3. 30 min−2 h: sitting up 4. 30 min−2 h: lying on your left side; then back to position #1 5. repeat the cycles as many times as possible.
			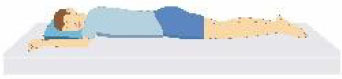
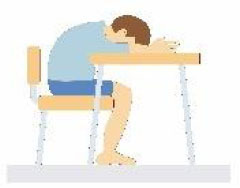	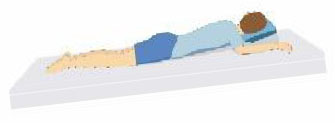	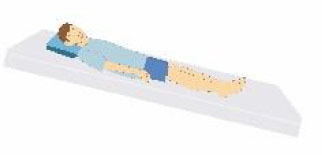	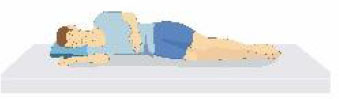
			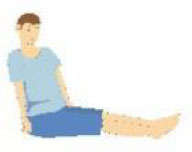
			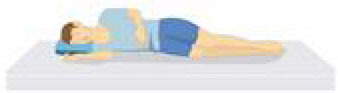
			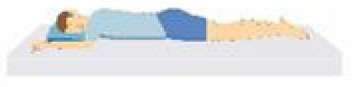

The guidance of ICS (9) suggested that cooperative, confirmed COVID-19 patients with FiO2 ≥ 28% or requiring basic respiratory support to achieve a SaO2 of 92–96% (88–92% if a risk of hypercapnic respiratory failure) are suitable candidates for awake-proning. Even though this criterion can be appropriately extended to mild to moderate ARDS, patients with severe ARDS (PaO2/FiO2 <100) are unsuitable candidates due to the risk of delayed intubation and subsequent treatment failure. Nonetheless, awake-proning is the optimal choice if the thoracic CT scan imaging shows significant dorsal consolidation. Without predominant dorsal consolidation, even with refractory hypoxemia, the prone position is not considered beneficial. Additionally, C-ARDS patients with respiratory distress, immediate need for intubation, hemodynamic instability (SBP <90 mmHg), arrhythmia, agitation or altered mental status, unstable spine, thoracic injury, recent abdominal surgery, facial injury, neurological issues, morbid obesity, or pregnancy (2/3rd trimesters) are also inadequate candidates. Furthermore, identifying pragmatic surrogate markers to predict the disease phase may be useful in selecting those who could benefit the most from awake-prone positioning. Coppo et al. (33) posited that patients are more likely to respond to prone positioning if performed early, following hospital admission, and in patients with increased inflammatory markers, such as increased lactate dehydrogenase or C-reactive protein concentrations. Moreover, the soluble receptor for advanced glycation end products (sRAGE) was identified as a biomarker of COVID-19 disease severity and an indicator of the need for mechanical ventilation, ARDS, and mortality. The combination of sRAGE and cytokine (e.g., IL-6) levels improved the prediction for MV use in inpatients (57). These studies indicated that the combination of different biomarkers for alveolar damage, lung inflammatory response, and lung compliance may be useful to determine individualized strategies for C-ARDS patients. An initial trial of the prone position may effectively distinguish patients tolerating prone positioning, improve hypoxemia, decrease the risk of P-SILI, and avoid an excessive intubation delay.

Some investigators have postulated that the clinical features of C-ARDS do not completely equate to typical ARDS. This implies that the experience obtained from typical ARDS cannot be fully applied to C-ARDS. Furthermore, this finding is supported by the research of Gattinoni et al. (15), who hypothesized that COVID-19 is a specific time-dependent disease with different responses to prone positioning. In the early phase of C-ARDS, lung compliance has been proposed to be high due to a loss of lung perfusion regulation and hypoxemic vasoconstriction. Since relatively high compliance indicates a well-aerated lung in C-ARDS, awake-prone positioning may not be effective. As the disease evolves, C-ARDS gradually manifests as typical ARDS with low lung compliance and responds to awake-prone positioning. The author identified Type L and Type H patients through CT scan imaging. However, this classification of COVID-19 needs to be further investigated. It may, to some degree, clarify why the C-ARDS patients sometimes do not respond to awake-proning. Hence, it is imperative to choose the appropriate treatment (endotracheal intubation) in a timely manner.

While there are no set criteria for intubation, high-acuity patients found to be at the greatest risk for intubation are as follows: (i) Patients with rapid progression over hours, hypoxemia not ameliorating (SPO2 ≤ 93 %) on HFNC or NIV treatment, or excessive inhalation effort. (ii) Patients with evolving hypercapnia, increasing respiratory rate (RR≥35 beats/min), decreasing tidal volume, worsening mental status, increasing duration, and depth of desaturations. (iii) Patients with hemodynamic instability or multiorgan failure. (iv) Patients with a persistent need for high flows/fraction of inspired oxygen (e.g., >60 L/minute and FiO2 >0.6) (58).

## Conclusion

In summary, awake-prone positioning appears safe and is a simple strategy that can be employed in most circumstances. In this narrative review, it was revealed that awake-prone positioning could improve the oxygenation level and short-term prognosis in select patients with hypoxemic respiratory failure attributable to C-ARDS. Exercising awake-prone positioning will relieve the shortage of ventilators and lessen the burden of the -intensive care unit. Although the improvement in oxygenation may persuade clinicians to delay intubation and result in a worsened prognosis, awake-prone positioning can be regarded as an “adjunctive” rather than a “rescue” maneuver in C-ARDS. The high-acuity patients at the greatest risk for requiring intubation were also summarized. However, the effects on tracheal intubation and survival rates in awake-prone positioning remain uncertain. Hence, further studies are warranted to confirm the effects of early application of awake-prone positioning on respiratory drive and the long-term clinical efficacy, such as intubation and survival rates. Numerous ongoing clinical trials will address some of these questions (Supplementary Table 2) and may provide a greater understanding of patient selection, development of combining biomarkers, the optimal duration, and long-term clinical outcomes.

## Author Contributions

LC contributed to conceptualization, methodology, data collection, curation, formal analysis, visualization, and original draft writing. YZ, YL, CS, and FL contributed to methodology, formal analysis, visualization, writing review and editing, and supervision. PP contributed to conceptualization, methodology, formal analysis, visualization, writing original draft, and supervision. All the authors approved the final version of the manuscript to be published.

## Funding

This study was supported by the National Natural Science Foundation of China (No. 81770080) and the Key R&D Program of Hunan Province (No. 2022SK2038).

## Conflict of Interest

The authors declare that the research was conducted in the absence of any commercial or financial relationships that could be construed as a potential conflict of interest.

## Publisher's Note

All claims expressed in this article are solely those of the authors and do not necessarily represent those of their affiliated organizations, or those of the publisher, the editors and the reviewers. Any product that may be evaluated in this article, or claim that may be made by its manufacturer, is not guaranteed or endorsed by the publisher.
