# Supplementary material for: The Application of Awake-Prone Positioning Among Non-intubated Patients With COVID-19-Related ARDS: A Narrative Review
Source: Front Med (Lausanne). 2022 Feb 7;9:817689. doi: 10.3389/fmed.2022.817689 (PMC8858818; doi:10.3389/fmed.2022.817689)
Supplement: Supplementary file 1 [file Data_Sheet_1.docx]

**Supplementary Table 1.** **Summary table of the major studies performed on awake prone positioning in patients with COVID-19.**

| Author | Research Type | Patients  Number | Age | Setting | Mode of Oxygen  Therapy | Feasibility | Prone Positioning Protocol | Complications | Results |
| --- | --- | --- | --- | --- | --- | --- | --- | --- | --- |
| Ehrmann (39) et al. | randomized, controlled, multinational, open-label meta-trial | 1121 | mean age 61.5 years | ICU, ED, or ward | HFNC | not reported | as long as possible each day | skin breakdown: PP 8(1%) vs SP 10(2%), vomiting: PP 15(3%) vs SP 18(3%), and central or arterial line dislodgement: PP 26(5%) vs SP 17(3%), Cardiac arrest at any time: PP 3(1%) vs SP 1(0%) | Treatment failure occurred in 223 (40%) of 564 patients assigned to AP and in 257 (46%) of 557 patients assigned to standard care |
| Perez-Nieto (49) et al. | retrospective, observational study | 827 | mean age 54.3 years | ICU, ED, or ward | LFNC, NRM, HFNC | not reported | 12-15h | not reported | A lesser proportion of patients in the AP group required endotracheal intubation (23.6% vs 40.4%) or had a lethal outcome (19.8% vs 37.3%). |
| Ferrando (31) et al. | A multicenter, prospective, adjusted observational cohort study | 199 | mean age 60.3 years | ICU | HFNO | not reported | ≥16h/d regardless of the number of sessions | not reported | The intubation rate in the AP group was 19.8% vs 37.3% in the supine group |
| Rosén (29) et al. | multicenter randomized clinical trial | 141 | mean age 65 years | ICU or ward | HFNO or NIV | not reported | targeting at least 16 h/d, Prone, and semi-prone positioning | pressure sores: AP 2(6%) VS SP 9(23%), all located in the lower back or gluteal region | Within 30 days after enrollment, 13 patients (33%) were intubated in the control group versus 12 patients (33%) in the prone group |
| Coppo (33) et al. | prospective cohort study | 56 | mean age 57.4 years | outside of ICU | CPAP and COT | feasible in 47 patients, unfeasible in 9 patients | mean 3.5 days, per session >3h | discomfort worsening(n=5), coughing(n=1) | The AP group required endotracheal intubation (23.6% vs 40.4%) or had a lethal outcome |
| Caputo (36) et al. | observational cohort study | 50 | median age 59 years | ED | NRB | not reported | 5 minutes of prone | not reported | SpO2 improved to 94% (IQR 90 to 95);13(24%) failed to improve and intubated |
| Thompson (34) et al. | single-center cohort study | 29 | mean age 67 years | step-down unit | HFNC or NRB | 25 had at least 1 awake session lasting> 1 hour;4 intubated | as long as tolerated up to 24h daily | not reported | SpO2 increased compared with baseline; 7 (37%) required intubation |
| Elharrar (32) et al. | A prospective, single-center, before-after study | 24 | mean age 66.1 years | outside of ICU | HFNC or COT | 4 did not tolerate PP≥1h, and 15 tolerated≥3h | ≥3h | Back pain AP:10(42%) | 6 patients were responders to PP, oxygenation increased during PP by only 25% and was not sustained in half of those after resupination |
| Winearls (50) et al. | retrospective observational study | 24 | mean age 62 years | HCU | CPAP | 2 failed to tolerate PP: 1 worsening oxygenation and 1 pain | in the first 24h was 8±5h, continued for a mean of 10±5 days | Pain (n=1) | PaO2:FiO2 and ROX index improved |
| Cammarota (30) et al. | prospective observational study | 20 | mean age 65 years | ICU | NIV | not reported | 1h | not reported | SpO2 improvement [96 (94–97) % supine vs 98 (96–99) % prone, p=0.008], a worsening in comfort score from 7.0 (6.0–8.0) to 6.0 (5.0–7.0) (p=0.012) and an increase in diaphragmatic thickening  fraction from 33.3 (25.7–40.5) % to 41.5 (29.8–50.0) % (p=0.025) |
| Xu (37) et al. | retrospective observational study | 10 | mean age 50.2 years | not reported | HFNC | all 10 patients can tolerate | more than 16 h per day and can be shortened by the patient’s tolerance | Not reported | median PaCO2 increases slightly [32.3(29.3–34.0) vs. 29.7 (28.0–32.0), p< 0.001]; PaO2/FiO2 elevated significantly; None of the patients progressed or intubation. |
| Ng (38) et al. | retrospective observational study | 10 | mean age 60 years | general ward | COT | All 10 patients were able to tolerate | 1h each session, 5 sessions a day | musculoskeletal discomfort, nausea, or vomiting. | 3 patients were transferred to ICU due to increased oxygen requirements, 1 patient was intubated and died, 9 patients weaning off oxygen |
| Sartini (35) et al. | cross-sectional survey | 15 | mean age 59 years | Outside of ICU | NIV | not reported | 3h (IQR, 1-6h) | not reported | RR decrease, SpO2, and PaO2:FIO2 improve, at 14-day, 9 patients were discharged, 1 improved and stopped pronation, 3 continued pronation, 1 patient was intubated and 1 patient died |
| Despres (59) et al. | retrospective observational study | 16 | mean age 60 years | not reported | HFNC or HFNO/COT | not reported | 9 PP sessions | not reported | The PaO2/FiO2 ratio improved after 4 sessions; Intubation was avoided in 3 patients |
| Singh (51) et al. | retrospective observational study | 15 | mean age 51.5 years | HDU | facemask, NRM, NIV | both tolerated | 10–12h/day, mean 11 days | not reported | the mean PaO2/FiO2 ratio increase 37.8 mmHg (p = 0.005),2 intubated. |
| Solverson (23) et al. | historical cohort study | 17 | mean age 54 years | ICU or general ward | HFNC, NRM | Eight patients (47%) had no tolerability problems | median 2 days and the median 2 sessions/day, duration 75 min | pain/general discomfort (47%) and delirium (6%) | improvements in oxygenation and RR,7 (41%) intubated, and 2 (12%) died. |
| Tu (60) et al. | retrospective observational study | 9 | mean age 51 years | not reported | HFNC | not reported | median 5 procedures per subject (twice daily) and median 2h | not observed | SpO2 increased from 90%±2% to 96%±3%; mean PaCO2 decreased from 47±7 to 39±5 mmHg;2 patients intubated |
| Taboada (61) et al. | prospective observational study | 7 | mean age 65 years | ICU | not reported | both tolerated | A total of 16 PP sessions and the median duration was 10h | not reported | PaO2/FiO2 increased,2 intubated, all 7 discharged from the ICU |
| Jayakumar (52) et al. | multi-center randomized controlled study | 60 | mean age 54.8-57.3 years; | not reported | facemask, NRM and NIV, HFNC | in PP group,43% (13/30) tolerated for 6h or more,70% tolerated for 4 hours | prone for a minimum of 6h/d, followed for 7 days | not reported | no significant difference in the cumulative fluid balance, length of stay, respiratory escalation, other medications use or mortality between the groups |
| Oliveira (62) et al. | prospective observational study | 41 | mean age 54.1 years | ICU | HFNC, NIV, a reservoir mask, or a nasal cannula | not reported | not available | not reported | Responders showed increased SpO2 (P < .001), PaO2 (P < .001), and PaO2 /FIO2 ratios (P < .001) with the maneuver and reduced breathing frequency, shorter lengths of stay in the ICU (P < .001) and hospital (P < .003), lower intubation rates at 48 h (P < .012), fewer days of ventilation (P < .02), and lower mortality (P < .001). |
| Taylor (63) et al | a cluster randomized pilot trial | 40 | mean age 50-60 years | referral center | LFNC, MFNC, HFNC, BiPAP | intolerance by 4 of 6 patients, most nursing staff deemed it not feasible | prone position as long as possible, allowed to return to the supine position | not reported | S/F ratio: UC group (n = 13) 216 VS APP group (n=27) 253; 48-hour study period with an S/F ratio below 315: UC group 42h VS APP group 20h |
| Taboada (64) et al. | prospective observational study | 63 | mean age 67 years | ICU | HFNO | not reported | Prone positioning was applied with a median of 4 (IQR: 2.5–8) sessions per subject. | not reported | No control group, among 63 patients, 43 (68.3%) were weaned from HFNO, 7 (11.1%) died, and 6 (9.5%) remain in ICU. 19 (30.2%) patients required intubation. 49 (77.8%) patients were discharged from the ICU |
| Hallifax (65) et al. | single-center retrospective study | 48 | mean age 69 years | HDU | CPAP, HFNO | 41% patients tolerate | prone or semi-prone position as tolerated for periods of ≥2 hours at least twice daily | not reported | full proning mortality 0/11, 0.0%; non-full proning mortality 12/19, 63.2%,p=0.003; Successful proning was associated with reduced odds of death (OR 0.06) (95% CI 0.01 to 0.55) |
| Bastoni (66) et al. | prospective observational study | 10 | mean age 73 years | ED | CPAP | 6 patients of 10 completed a cycle of prone ventilation. | As long as possible | not reported | After 1 hour of proning, improvement in PaO2/FiO2 ratio for all the patients (median 97±8 mm Hg). |
| Dubosh (67) et al. | prospective, observational cohort study | 22 | mean age 61 years | ED | not reported | only 1 patient cannot tolerate | patients completed at least 30 min of proning | None of the patients experienced immediate vomiting, respiratory decompensation, immediate need for intubation, or death upon proning | SpO2:FiO2 ratio increased in minutes 5–35 of proning compared to the 5 min;7 patients intubated;2 patients died and 20 (91%) survived to hospital discharge. |
| Avdeev (68) et al. | prospective cohort study | 22 | mean age 48.5 years | care units | CPAP, oxygen therapy | not reported | not reported | not reported | 16 of 22 patients (72.7%) responded to PP treatment with increase in PaO2/FiO2, RR also improved; The patients who responded to PP had disturbances of aeration in posterior regions (8.5 (7.3–9.8) vs. 6.0 (4.3–7.3); p = 0.006) as reflected by greater LUS. |

Abbreviations: ICU: Intensive Care Unit; ED: Emergency Department; LFNC: Low-Flow Nasal Cannula; NRM: Non-Rebreather Mask; MFNC: Medium-Flow Nasal Cannula; HFNC: High-Flow Nasal Cannula; HFNO: High-Flow Nasal Oxygen; NIV: Noninvasive Ventilation; COT: Conventional Oxygen Therapy; CPAP: Continuous Positive Airway Pressure; HCU: high care unit; HDU: high-dependency unit, ROX: ratio of oxygen saturation as measured by pulse oximetry/FiO2 to respiratory rate; RR: Relative Risk; PaO2/FiO2: Partial Pressure of Oxygen in Arterial Blood/ Fraction of Inspired Oxygen; SpO2: Saturation of Peripheral Oxygen.

**Supplementary Table 2.** **Registries of ongoing clinical trials of awake prone positioning in patients with COVID-19.**

| No. | Main ID | Public Title | Registration Date | Last Refreshed date | Recruiting Status | Prospective Registration |
| --- | --- | --- | --- | --- | --- | --- |
| 1 | NCT05083130 | Awake Prone Positioning in Moderate to Severe COVID-19 | 2021/10/10 | 26 October 2021 | Not recruiting | Yes |
| 2 | CTRI/2021/09/036606 | Finding out the effect of oxygen therapy in awake prone position in patient with covid pneumonia in tertiary care hospital | 2021/9/17 | 24 November 2021 | Not Recruiting | Yes |
| 3 | NCT05060926 | Intubation Prediction in COVID-19 Patients Treated With Awake Prone Positioning | 2021/9/16 | 11 October 2021 | Recruiting | Yes |
| 4 | IRCT20210724051970N1 | Evaluation of prone positioning in improving breathing of awake patients with dyspnea due to COVID-19 | 2021/9/3 | 1 November 2021 | Recruiting | Yes |
| 5 | NCT04982341 | HFNC and Prone Positioning in Awake Patients With Severe COVID-19 | 2021/7/19 | 10 August 2021 | Not recruiting | No |
| 6 | CTRI/2021/06/034340 | Impact of reinforced awake proning on outcomes of COVID-19 pneumonia | 2021/6/22 | 24 November 2021 | Not Recruiting | Yes |
| 7 | NCT04924816 | Awake Prone Position in Patients With COVID-19 | 2021/6/10 | 21 June 2021 | Not recruiting | No |
| 8 | CTRI/2021/05/033869 | Can the benefit of prone position benefit in reducing oxygen requirements in oxygen-dependent COVID-19 patients | 2021/5/31 | 24 November 2021 | Recruiting | Yes |
| 9 | NCT04853979 | Awake Prone Positioning in COVID-19 Suspects With Hypoxemic Respiratory Failure | 2021/4/12 | 21 June 2021 | Recruiting | Yes |
| 10 | CTRI/2021/04/032501 | Comparison of effect of High Flow Nasal Cannula with Continuous Positive Airway Pressure in reducing incidence of invasive mechanical ventilation in severe COVID 19 patients. | 2021/4/1 | 24 November 2021 | Not Recruiting | Yes |
| 11 | NCT04760561 | Self-prone Positioning for Awake Non-intubated Patients With COVID-19 | 2021/2/15 | 1 March 2021 | Not recruiting | Yes |
| 12 | NCT04667286 | Awake Pronation for Covid-19 Treatment | 2020/12/11 | 26 April 2021 | Recruiting | Yes |
| 13 | CTRI/2020/12/029587 | To compare the effect of lying in face-down position along with oxygen therapy given through high flow nasal cannula device versus lying face down with oxygen given by non-rebreathing face mask in COVID-19 patients. | 2020/12/4 | 24 November 2021 | Not Recruiting | Yes |
| 14 | NCT04649658 | Pronation in COVID-19 Patients Undergoing Non-Invasive Respiratory Support | 2020/11/30 | 11 January 2021 | Not recruiting | Yes |
| 15 | CTRI/2020/11/029130 | The lying in prone position effect on oxygen saturation in patients in early-stage and not intubated having mild to moderate Lung Disease with COVID-19 | 2020/11/16 | 24 November 2021 | Not Recruiting | Yes |
| 16 | NCT04632602 | Awake Prone Position to Reduce Ventilation Inhomogeneity in COVID-19 Acute Respiratory Failure | 2020/11/16 | 24 August 2021 | Not recruiting | Yes |
| 17 | NCT04547283 | Awake-Prone Positioning Strategy for Hypoxic Patients With COVID-19 | 2020/9/9 | 12 July 2021 | Not recruiting | Yes |
| 18 | CTRI/2020/09/027562 | Effect of prone positioning with HFNC on oxygenation and overall outcomes in severe COVID-19 patients. | 2020/9/3 | 24 November 2021 | Not Recruiting | Yes |
| 19 | RBR-2k66ft | Randomized, study to evaluate early prone position in patients with COVID-19 | 2020/8/10 | 6 December 2021 | Not Recruiting | Yes |
| 20 | NCT04477655 | Prone Positioning in Non-intubated Patients With COVID-19 Associated Acute Respiratory Failure | 2020/7/15 | 26 April 2021 | Not recruiting | Yes |
| 21 | CTRI/2020/07/026532 | Comparison to two different position to relieve difficulty in breathing in COVID- 19 patients | 2020/7/13 | 24 November 2021 | Not Recruiting | Yes |
| 22 | CTRI/2020/07/026349 | Effect of awake proning in patients with COVID-19 related respiratory failure | 2020/7/3 | 24 November 2021 | Recruiting | Yes |
| 23 | ISRCTN54917435 | Could prone positioning increase lung function for patients with severe COVID-19 infection? | 2020/6/15 | 24 August 2021 | Not Recruiting | Yes |
| 24 | CTRI/2020/06/025804 | Effectiveness of lying face down in improving the outcome of COVID-19 patients | 2020/6/11 | 24 November 2021 | Not Recruiting | Yes |
| 25 | NCT04407468 | Awake Prone Positioning and Oxygen Therapy in Patients With COVID-19 | 2020/5/27 | 12 December 2020 | Not recruiting | Yes |
| 26 | NCT04395144 | COVid-19: Awake Proning and High-flow Nasal Cannula in Respiratory Distress | 2020/5/15 | 29 March 2021 | Not recruiting | Yes |
| 27 | NCT04368000 | Prone Positioning in Awake Patients With COVID-19 Requiring Hospitalization | 2020/4/24 | 11 January 2021 | Not recruiting | Yes |
| 28 | NCT04350723 | Awake Prone Position in Hypoxemic Patients with Coronavirus Disease 19 COVID-19 (COVI-PRONE) | 2020/4/14 | 26 October 2021 | Not recruiting | Yes |
| 29 | NCT04344587 | Awake Prone Position for Early Hypoxemia in COVID-19 | 2020/4/8 | 8 November 2021 | Not recruiting | Yes |
| 30 | NCT04347941 | Awake Prone Positioning to Reduce Invasive Ventilation in COVID-19 Induced Acute Respiratory Failure | 2020/4/8 | 6 December 2021 | Not recruiting | Yes |
| 31 | UMIN000045889 | Adaptive, multicenter, randomized, controlled, open trial of the use of early prone positioning in conscious patients with severe COVID-19 without invasive ventilatory support, to prevent invasive mechanical ventilation and death. | 2021/10/27 | 28 October 2021 | Recruiting | Yes |

*Data from the international registries are recognized by the WHO (https://www.who.int/clinical-trials-registry-platform/network/primary-registries) and UMIN-CTR (https://www.umin.ac.jp/ctr/).
